# Inositol Polyphosphate-4-Phosphatase Type I Negatively Regulates Phagocytosis via Dephosphorylation of Phagosomal PtdIns(3,4)P_2_

**DOI:** 10.1371/journal.pone.0142091

**Published:** 2015-11-04

**Authors:** Kiyomi Nigorikawa, Kaoru Hazeki, Junko Sasaki, Yumio Omori, Mikiko Miyake, Shin Morioka, Ying Guo, Takehiko Sasaki, Osamu Hazeki

**Affiliations:** 1 Graduate School of Biomedical & Health Sciences, Hiroshima University, Hiroshima 734–8553, Japan; 2 Department of Pathology and Immunology, Akita University School of Medicine, Akita 010–8543, Japan; The Ohio State University, UNITED STATES

## Abstract

Phagocytosis is a highly conserved process whereby phagocytic cells engulf pathogens and apoptotic bodies. The present study focused on the role of inositol polyphosphate-4-phosphatase type I (Inpp4a) in phagocytosis. Raw264.7 cells that express shRNA against Inpp4a (shInpp4a cells) showed significantly increased phagocytic activity. The introduction of shRNA-resistant human Inpp4a abolished this increase. Macrophages from Inpp4a knockout mice showed similar increases in the phagocytic activity. Inpp4a was recruited to the phagosome membrane by a mechanism other than the direct interaction with Rab5. PtdIns(3,4)P_2_ increased on the phagosome of shInpp4a cells, while PtdIns(3)P significantly decreased. The results indicate that Inpp4a negatively regulates the phagocytic activity of macrophages as a member of the sequential dephosphorylation system that metabolizes phagosomal PtdIns(3,4,5)P_3_ to PtdIns(3)P.

## Introduction

Endocytosis (phagocytosis and pinocytosis) is a plasma membrane invagination and scission process that leads to the formation of intracellular carrier vesicles. Phagocytosis occurs in professional phagocytes, such as macrophages, neutrophils and dendritic cells, and is used to take up solid particles. Pinocytosis occurs in all cells that engulf fluid and solutes. The process of phagocytosis involves specific cell-surface receptors. One group of receptors, including mannose receptors and Toll-like receptors (TLRs), directly interacts with pathogens by recognizing the conserved motifs of microorganisms. Another group of receptors recognizes opsonins, which are host molecules (e.g., antibodies and complement components) that interact with the surface molecules of invaders [1]. Pinocytosis occurs via at least four basic mechanisms: macropinocytosis, clathrin-mediated endocytosis, caveolin-mediated endocytosis, and clathrin- and caveolin-independent endocytosis [2].

Several phosphoinositide (PI) species play inevitable roles in the endocytic process [3, 4]. A rapid turnover of PtdIns(4,5)P_2_ is required for the early steps of endocytosis to progress [5]. A transient and dramatic increase in PtdIns(3,4,5)P_3_ on the phagocytic cup is necessary for phagosome formation [5]. However, the fluctuation of PtdIns(3,4,5)P_3_ is unnecessary for the clathrin-mediated pinocytosis of soluble ligands [5, 6]. Following scission from the plasma membrane, both a nascent phagosome and pinosome fuse with a common compartment, an early endosome, on which PtdIns(3)P recruits Rab5 effectors, such as EEA1 (early endosome antigen 1), that are required for the proper maturation of the endosomes [7, 8]. The presence of a variety of PI-metabolizing enzymes that may enable the precise adjustment of the different endocytic processes has been described [5, 9].

Two types of inositol polyphosphate-4-phosphatase, type I (Inpp4a) and type II (Inpp4b), have been identified in mammals [10]. Inpp4a and Inpp4b use PtdIns(3,4)P_2_ as a preferential substrate and produce PtdIns(3)P [10]. Inpp4a has been found on early and recycling endosomes [10]. The RNAi-based knock down of the isozyme reduces the endocytosis of transferrin in Hela cells [11]. The reduced internalization of cell surface N-methyl-D-aspartate-type glutamate receptors (NMDARs) in the striatum of Inpp4a knockout mice also demonstrates the role of Inpp4a as a positive regulator of clathrin-mediated endocytosis [12]. Although little is known about the role of Inpp4b in endocytosis, the depletion of the isozyme with siRNA reportedly impairs the dextran uptake in A431 cells [13]. Contrastingly, the transferrin uptake in Cos7 cells is inhibited by the overexpression of the membrane-anchoring form of Inpp4b [14].

In the present study, we focused on the role of Inpp4a in phagocytosis. Inpp4a was found to negatively control phagocytosis, which opposes its role as a positive regulator of clathrin-mediated endocytosis. A spatiotemporal analysis of the localization of PIs and Inpp4a with fluorescent probes showed that Inpp4a is recruited to the phagosome by a Rab5-independent mechanism and functions as a member of the sequential dephosphorylation system that metabolizes phagosomal PtdIns(3,4,5)P_3_.

## Materials and Methods

### Materials

The materials were obtained from the following sources: zymosan, fluorescein isothiocyanate (FITC)-zymosan, zymosan opsonizing reagent, LysoTracker, RPMI 1640 medium (Life Technologies Co., Carlsbad, CA, USA); sheep red blood cells (RBCs) (Cosmo Bio Co., Tokyo, Japan); Alexa488-conjugated anti-rabbit IgG (Cell Signaling, Danvers, MA, USA); protein assay kit (Bio-Rad, Hercules, CA, USA); anti-SRBC (InterCell Technologies, Jupiter, FL, USA); anti-Inpp4a was prepared as previously [12]; Na_2_^51^CrO_4_ (MP Biomedicals).

### Mice

This study was carried out in accordance with the NIH Guide for Care and Use of Laboratory Animals and approved by the animal care and use committee at Hiroshima University (Permit number: A13-151). All efforts were made to minimize suffering. Inpp4a^flox/flox^ mice were prepared as previously reported [12]. Inpp4a^flox/flox^ mice were crossed with transgenic mice expressing Cre under the control of the CD11b promoter. Mice were maintained at 22°C in a 12-h light/dark cycle, and used between 8 and 12 weeks after birth. All the mice were anesthetized with diethyl ether and then decapitated before harvest of peritoneal cells or heart. Peritoneal macrophages were prepared as described previously [15]. Briefly, mice were injected (i.p.) with 2 mL of 3% thioglycollate broth under ether anesthesia. After 3 days, the peritoneal exudate was harvested by washing the peritoneal cavity with ice-cold phosphate-buffered saline (PBS). Cells were seeded (about 5 × 10^5^ cells/well) in 24-well plates and allowed to adhere to dishes by placing in an atmosphere of humidified 5% CO_2_ at 37°C for 1–2 h in RPMI 1640 medium supplemented with 10% fetal calf serum (FCS). Non-adherent cells were washed off with PBS and attached cells were designated as macrophages. Heart was harvested from the wild type mouse and about 0.1 g of the slice was homogenized in 1 ml of Sepasol RNA I Super G^TM^ (Nacalai Tesque, Kyoto, Japan) using Polytron homogenizer. Total RNA was prepared according to manufactures' protocol (Nacalai Tesque, Kyoto, Japan).

### Cells

**Fig 1:**
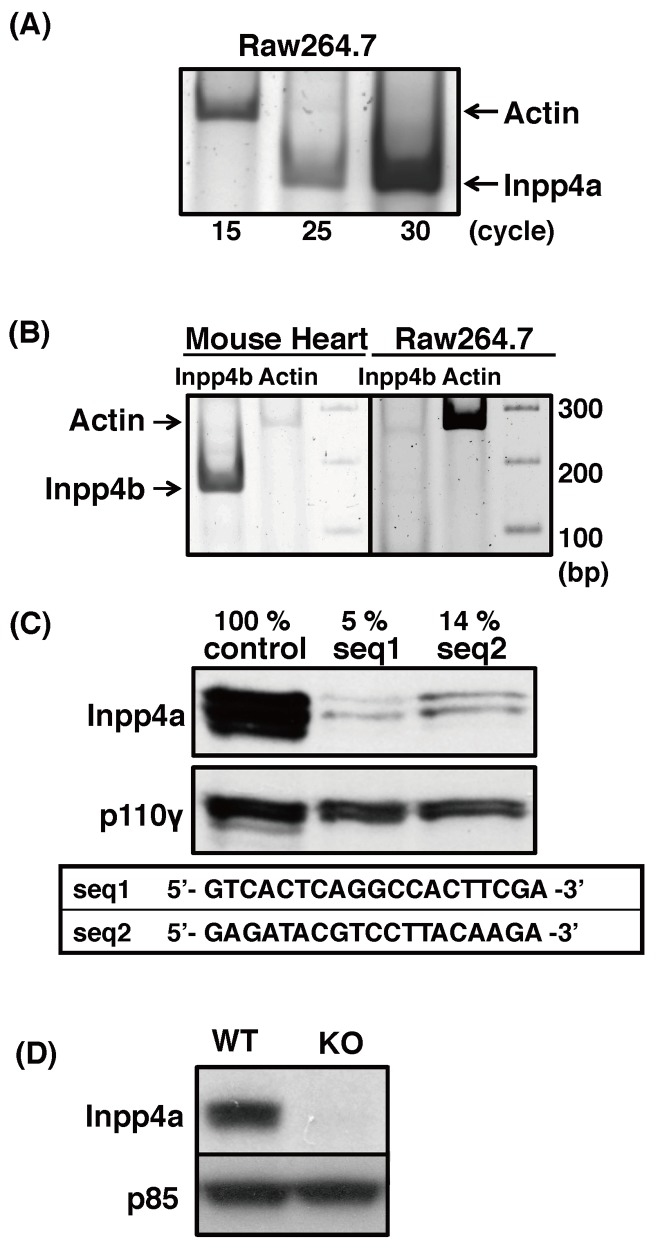
Preparation of Inpp4a-deficient cells. (A) Inpp4a mRNA was detected by RT-PCR with primer pairs shown in S1 Table. (B) Total RNAs from the mouse heart and Raw264.7 were subjected to RT-PCR. (C) Two lines of shInpp4a cells (seq1 and seq2) were prepared with the respective target sequences. The Inpp4a protein level was analyzed by western blotting. (D) Peritoneal macrophages were prepared from wild type (WT) or Inpp4a knockout (KO) mice. The Inpp4a protein level was determined by western blotting.

RAW264.7 cells (ATCC) lacking Inpp4a were prepared as follows: oligonucleotides targeting Inpp4a (Fig 1) were cloned into the pH1 vector downstream of the H1 RNA promoter as previously described [16, 17] to express siRNA (small interfering RNA) hairpins. For each of the targeted sequences, a pair of oligonucleotides was synthesized (Hokkaido Life Sciences, Sapporo, Japan) with the following sequences: 5′-CCC(X)_19_TTCAAGAGA(Y)_19_TTTTTGGAAA-3′ and 5′-CTAGTTTCCAAAAA(Y)_19_TCTCTTGAA(X)_19_GGGTGCA-3′, where (X)_19_ is the coding sequence and (Y)_19_ is the complementary sequence. The oligonucleotide pair was annealed and ligated downstream of the H1 RNA promoter at the PstI and XbaI sites of the pH1 vector. The vectors were transfected into RAW264.7 cells (5–10 × 10^6^ cells) at 250 V/950 μF (Gene Pulser II; Bio-Rad). Puromycin (3 μg/ml) was added to the cells 24 h after transfection, and the incubation was continued to select for resistant cells. To determine the efficiency of the gene silencing, the total RNA was isolated with Sepasol^TM^ (Nacalai Tesque, Kyoto, Japan), and the mRNA was quantified by reverse transcription PCR. The primer pairs used are listed on S1 Table. The expression of Inpp4a was determined by western blotting. Control cells were prepared as described above with a pH1 vector containing a 400-bp stuffer sequence instead of the target sequence. For the phagocytosis assay and the microscopic analysis, the cells were seeded in 24-well plates (Becton Dickinson) or tissue culture-coated glass bottom dishes (Greiner Bio-One) in RPMI 1640 medium containing 4.5 g/L glucose and 10% FCS in a humidified 5% CO_2_ atmosphere at 37°C. Immediately before starting the assay, the medium was aspirated and replaced with incubation buffer (complete RPMI 1640 medium without NaHCO_3_, fortified with 20 mM HEPES-NaOH, pH 7.4). The activities were then determined by incubating the cells at 37°C in a water bath at ambient conditions.

### Western blot

Cells in 24-well plate were washed with PBS and lysed in 50 μL of lysis buffer containing 25 mM Tris-HCl (pH 7.4), 0.5% Nonidet P-40, 150 mM NaCl, 1 mM sodium orthovanadate (Na_3_VO_4_), 1 mM EDTA, 0.1% BSA, 20 mM sodium fluoride, 1 mM phenylmethylsulfonyl fluoride, 2 μM leupeptin, 20 μM p-amidinophenylmethylsulfonyl fluoride, and 1 mM dithiothreitol. The cell lysates were centrifuged at 15,000 rpm for 10 min. Supernatants were collected, and the protein concentration was determined using the Bio-Rad assay kit. Total cell lysates (100 μg protein) were mixed with 10 μL of 5x sample buffer (62.5 mM Tris (pH 6.8), 1% SDS, 10% glycerol, 5% 2-mercaptoethanol, and 0.02% bromophenol blue) and heated at 100°C for 5 min. The proteins were separated by SDS-PAGE and transferred electrophoretically onto a polyvinylidene difluoride (PVDF) membrane (Millipore). The membrane was blocked with 5% skim milk and incubated with the appropriate antibodies. Antibody binding was detected using a chemiluminescent substrate (Perkin-Elmer).

### Preparation of IgG-coated RBCs and measurement of phagocytosis

The RBCs were labeled with ^51^Cr as described previously [15]. IgG-coated RBCs (E-IgG) were prepared by incubating labeled cells with rabbit anti-SRBC antibody at 37°C for 10 min in 5 mM veronal buffer (pH 7.5) supplemented with 0.1% gelatin, 75 mM NaCl, 0.15 mM CaCl_2_, 0.5 mM MgCl_2_, and 10 mM EDTA, followed by incubation on ice for 15 min. The E-IgG were washed thrice with 5 mM veronal buffer (pH 7.5) supplemented with 0.1% gelatin, 75 mM NaCl, 0.15 mM CaCl_2_, and 0.5 mM MgCl_2_ (GVB) and finally suspended in incubation buffer (detailed above). The binding and phagocytosis of E-IgG were measured as reported previously [15]. Monolayers of RAW264.7 cells (2 × 10^5^ cells/well in a 24-well plate) were incubated with ^51^Cr-labeled E-IgG (2 × 10^7^ cells) at 37°C for the indicated periods of time. The monolayers were then washed thrice with PBS to remove unbound E-IgG and then briefly exposed to 0.1 ml of hypotonic PBS (five-fold diluted). The radioactivity released into the supernatant during the hypotonic shock was measured as a proxy for the amount of E-IgG bound to the surface of the phagocytes. The monolayers were washed an additional three times with PBS and finally solubilized in 0.5% Triton X-100. The radioactivity of the solution was measured to determine the amount of engulfed E-IgG.

E-IgG binding was determined by Rosetting assay. Macrophages were incubated with E-IgG at 4°C for 30 min, washed thrice with ice-cold PBS, fixed with PBS containing 4% formaldehyde for 15 min and subjected to immune-staining with Alexa488-anti-rabbit IgG to easily count the E-IgG. After nuclearstain with DAPI, fluorescence images and phase-contrast images of at least 100 macrophages from three randomly selected fields were taken using confocal microscopy. The mean numbers of E-IgG binding to macrophages from three independent experiments are expressed as E-IgG/100 RAW264.7 cell.

The rescue experiment with shRNA-resistant Inpp4a was performed as follows. Cells were transfected with EGFP-Inpp4a (human origin) or EGFP and seeded into tissue culture-coated glass bottom dishes 24 h before the phagocytosis assay. The cells were added with E-IgG and centrifuged at 3,000 rpm for 1 min, followed by an incubation at 37°C for 15 min. The cells were then washed thrice with PBS, fixed with PBS containing 4% formaldehyde for 15 min at room temperature, permeabilized with PBS containing 0.3% Triton X-100 and 0.5% BSA for 60 min and incubated with Alexa647-anti-rabbit IgG. The number of engulfed E-IgG by EGFP-Inpp4a- or EGFP-transfected cells was determined using a BZ-H2C analysis system (Keyence, Osaka, Japan).

### Phagocytosis of zymosan

FITC-labeled zymosan particles were mixed with an equal amount of unlabeled zymosan to expedite counting, followed by sonication for approximately 1 min. The particles were then opsonized with anti-zymosan IgG in EDTA-GVB at 37°C for 60 min before use. The opsonized zymosan was washed thrice with GVB and finally suspended in incubation buffer. This prepared zymosan was sonicated for approximately 10 sec immediately before use. RAW264.7 cells (5 x 10^5^ cells/well in a glass bottom dish) were incubated at 37°C for the indicated durations with 5 x 10^6^ zymosan particles. Phagocytosis was stopped via the addition of ice-cold PBS. The cells were then washed thrice with PBS, fixed with 4% paraformaldehyde for 15 min at room temperature and finally rinsed with PBS. Fluorescence images (excitation 488 nm, emission 525 nm) and phase-contrast images of at least 100 macrophages from three randomly selected fields were taken using confocal microscopy. The mean numbers of ingested zymosan particles are expressed as particles/100 RAW264.7 cell.

### Plasmids

EGFP-[3×FYVE(EEA1)], EGFP-[2×PH(TAPP1)], EGFP-[PH(Akt)], EGFP-[PH1-PH2-PH1(MyoX)] [18, 19] and EGFP-Rab5b were prepared as previously described [20]. cDNAs encoding human INPP4A (GenBank accession number NM_030266.3) were amplified by reverse transcriptase-PCR from ovary total RNA (Agilent Technologies, Santa Clara, CA, USA) using primers possessing additional nucleotide sequences convenient for subcloning.

5’- CTCGAGTCACAGCAAGAGAGCACAGC -3’ (for pEGFP-C1 vector)

5’- GAATTCACAGCAAGAGAGCACAGCCC -3’ (for pCMV5-mCherry)

5’- AAGCTTCACGTTTCAACTTTTCCGTAAG 3’ (for pEGFP-C1 and pCMV5-mCherry)

### Transfection

The plasmids were transfected with the Neon^TM^ transfection system (Invitrogen) according to the manufacturer’s protocol. Approximately 24 h after transfection, the cells were subjected to a microscopic analysis.

### Monitoring PI dynamics during the course of phagocytosis

The cells transfected with fluorescent probes were cultured in tissue culture-coated glass bottom dishes (Greiner Bio-One). Medium was then replaced with CO_2_-independent incubation buffer described above and cells were placed on the microscope stage (BIOREVO BZ-9000 microscope equipped with a CFI Plan Apo VC60xH oil immersion lens) maintained at 37°C in an ambient atmosphere. After the addition of E-IgG, the fluorescent images were collected every 1 min. The intensity of the phagosome-associated fluorescence was analyzed using a BZ-II analysis system (Keyence, Osaka, Japan). In the figures where the time courses of two probes are compared, the fluorescence intensity of each probe was shown as % of respective maximum value. Time zero in the figures indicates the time when the mCherry-Inpp4a fluorescence around E-IgG peaked.

## Results

### Inpp4a inhibits phagocytosis

**Fig 2:**
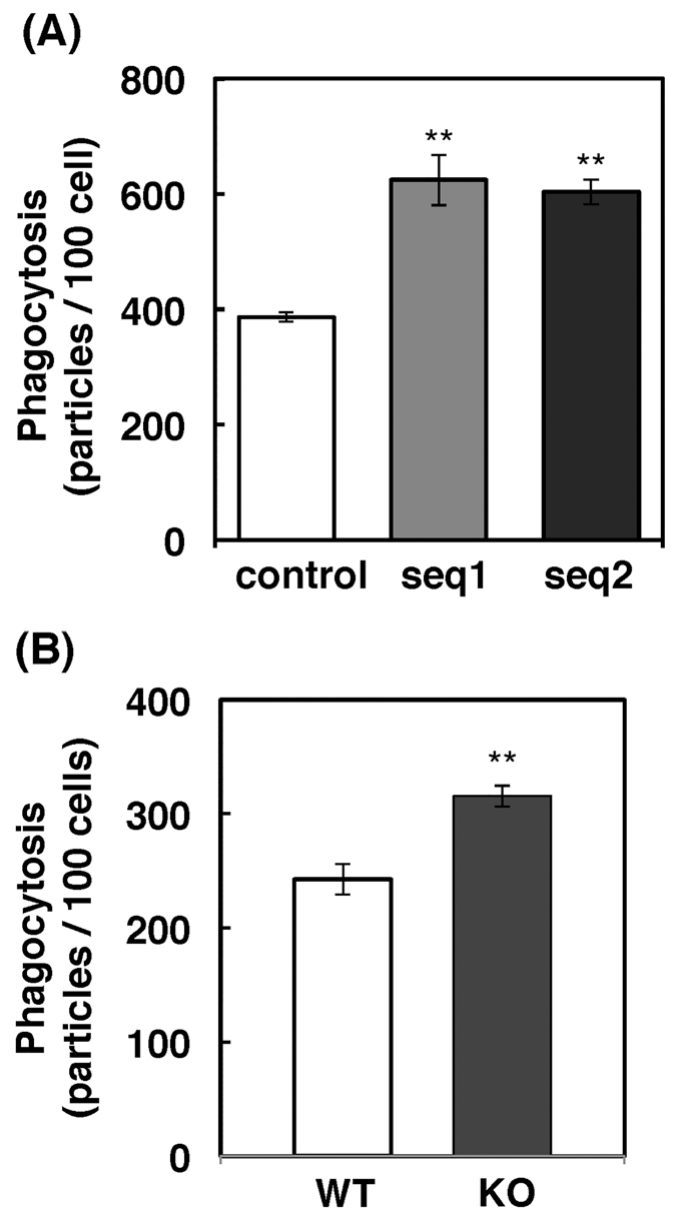
Enhanced phagocytosis of zymosan in Inpp4a-deficient cells. (A) Raw264.7 cells deficient in Inpp4a (seq1 and seq2) or control cells were incubated for 15 min with IgG-coated zymosan. (B) Peritoneal macrophages from wild type (WT) or Inpp4a knock out (KO) mice were incubated for 15 min with IgG-coated zymosan. (A, B) The number of fluorescent particles was counted in merged images, and the number of zymosan particles within the cells was calculated. The results from three separate experiments are shown as the means ± s.e.m. **P<0.01

We first determined the mRNA expression of Inpp4a and Inpp4b in Raw264.7 cells. Reverse transcriptase-PCR with specific primers showed that Inpp4a mRNA is expressed, while Inpp4b mRNA is not expressed in Raw264.7 cells (Fig 1A and 1B). The performance of the primer pair for Inpp4b mRNA was confirmed using cDNAs from the mouse heart (Fig 1B). Two lines of cells (shInpp4a-seq1 and shInpp4a-seq2 cells) that produce shRNAs targeting different Inpp4a sequences were prepared. The protein level of Inpp4a decreased to 5% and 14% of the wild type cells in seq1- and seq2-expressing cells, respectively (Fig 1C). The uptake of IgG-zymosan particles increased in the shInpp4a cells (Fig 2A). Binding of IgG-opsonized particles to FcγR is known to cause Akt phosphorylation as a result of cell accumulation of PtdIns(3,4,5)P_3_ and PtdIns(3,4)P_2_. The FcγR-induced Akt phosphorylation was markedly increased in the knockdown cells (S1A Fig). We next examined the phagocytic activity of macrophages from Inpp4a knockout mice, which were prepared by crossing Inpp4a^flox/flox^ mice with transgenic mice expressing Cre under the control of the CD11b promoter (Fig 1D). The macrophages from knockout mice showed increased uptake of IgG-opsonized zymosan (Fig 2B). FcγR-induced Akt phosphorylation was increased in the Inpp4a-deficient cells (S1B Fig).

**Fig 3:**
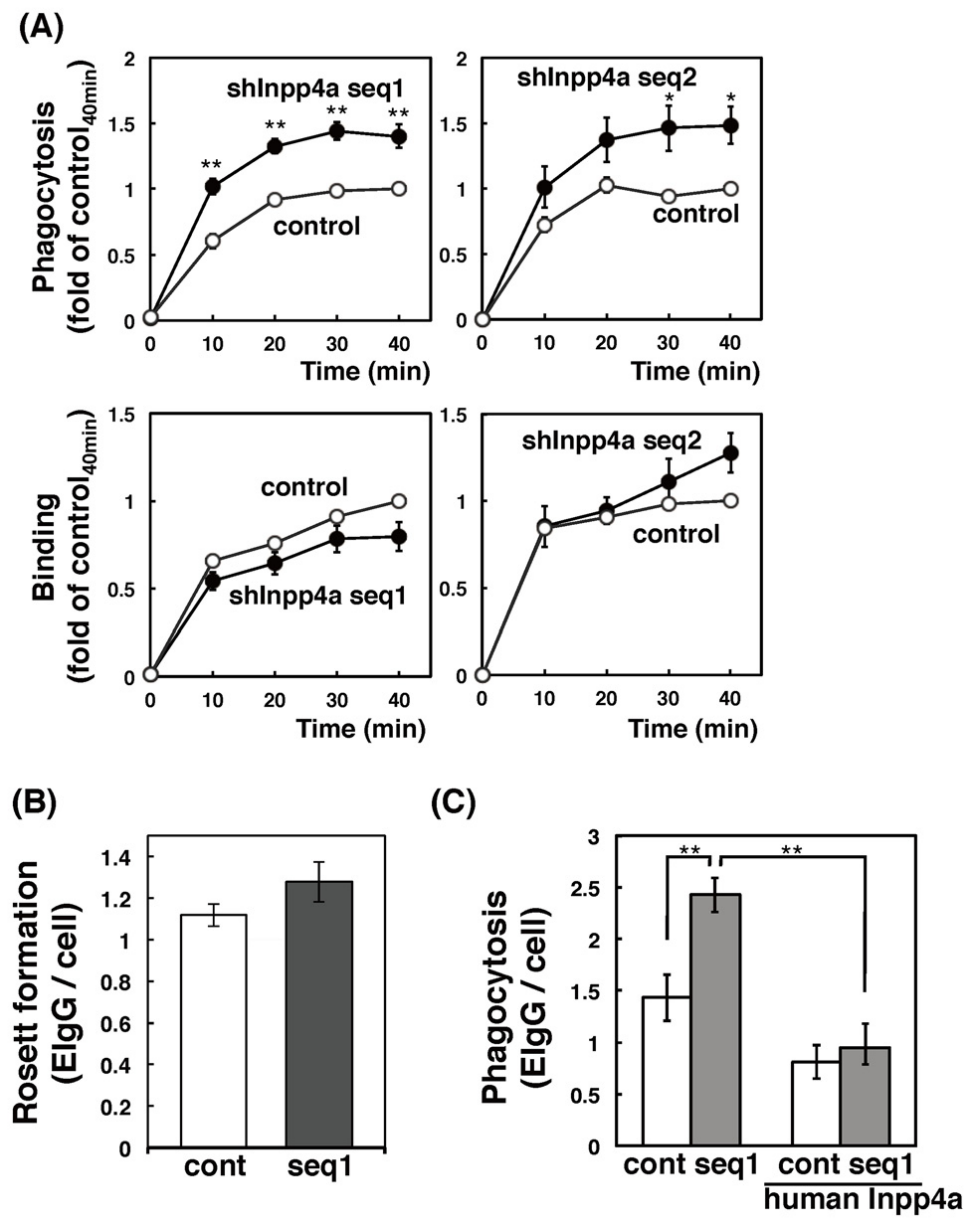
Enhanced phagocytosis of E-IgG in Inpp4a-deficient cells. (A) Control (open circles) or shInpp4a cells (closed circles) were incubated for the indicated times with E-IgG. The numbers of E-IgG on the outer surface of the cells (binding) and within the cells (phagocytosis) were determined as described in the Methods section. (B) Control (cont) or shInpp4a (seq1) cells were incubated on ice for 30 min with E-IgG. The bound E-IgG was visualized by staining with Alexa488-anti-rabbit IgG, and was counted under a microscope. (C) Control (cont) or shInpp4a (seq1) cells were transfected with EGFP-tagged human Inpp4a, cultured for 24 h, and added with E-IgG. The phagocytosis of the EGFP-expressing cells and non-expressing cells was determined under a microscope. The results from three separate experiments are shown as the means ± s.e.m.

We next tested the uptake of IgG-coated erythrocytes (E-IgG). The phagocytosis of E-IgG was significantly enhanced in both shInpp4a cells (Fig 3A, upper panels). The cell surface binding of E-IgG to the wild type and the knockdown cells was comparable (Fig 3A, lower panels). The result of Rosetting assay also indicated that the number of FcγR was unchanged (Fig 3B). When the shInpp4a cells were transfected with human Inpp4a, an shRNA-resistant Inpp4a, the level of phagocytosis was decreased to that in the wild type cells (Fig 3C). Thus, Inpp4a negatively regulates the FcγR-mediated phagocytosis.

### Inpp4a is involved in phagosomal PI metabolism

**Fig 4:**
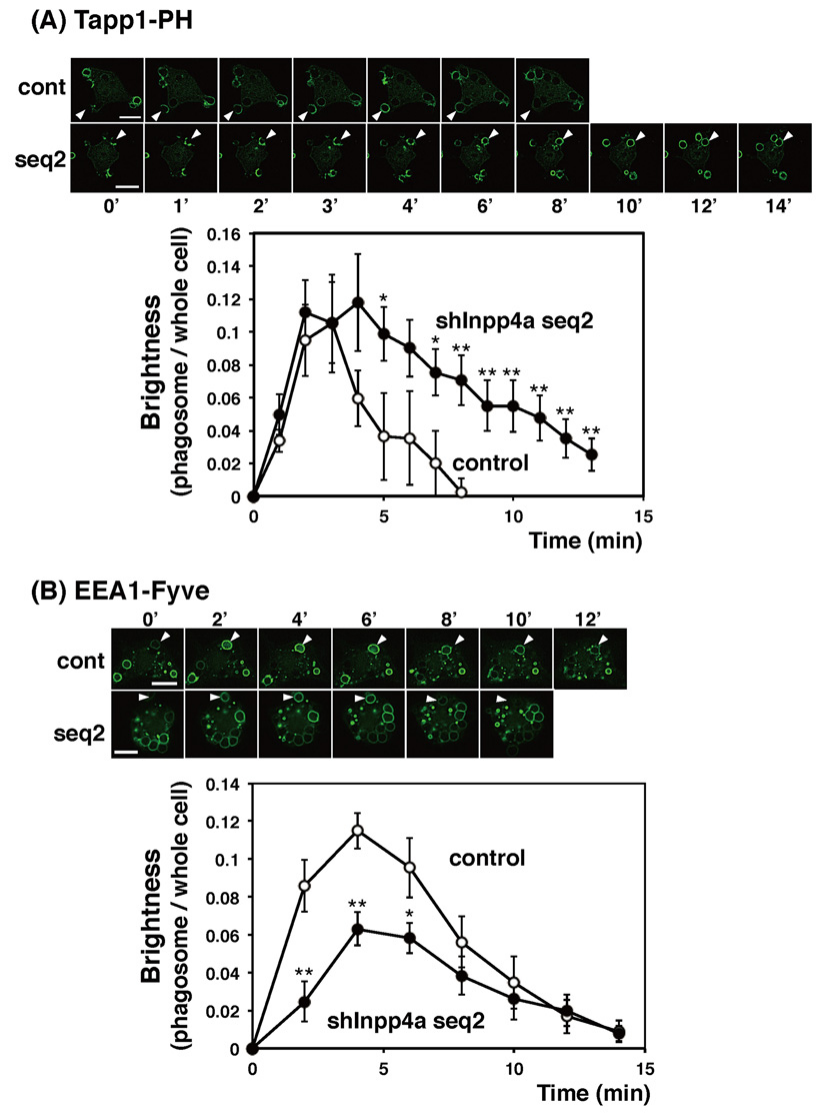
Phagosomal levels of PtdIns(3,4)P_2_ and PtdIns(3)P in Inpp4a-deficient cells. (A) Control (cont) or shInpp4a (seq2) cells were transfected with EGFP-[2×PH(TAPP1)]. (B) Control or shInpp4a (seq2) cells were transfected with EGFP-[3×FYVE(EEA1)]. (A, B; upper panels) the cells were challenged with E-IgG. The arrows indicate the phagosome. Bar, 10 μm. (A, B; lower panels) The intensity of the phagosome-associated fluorescence and total cellular fluorescence was quantified as described in the Methods section. The ratio of phagosome-associated fluorescence to total cellular fluorescence was calculated. The combined results from three separate cells are shown as the means ± s.e.m. *P<0.05, **P<0.01

We examined the dynamics of PtdIns(3,4)P_2_, the preferential substrate of Inpp4a, during the phagocytosis. In the experiment shown in Fig 4A, the cells were transfected with EGFP-[2×PH(TAPP1)], a specific probe for PtdIns(3,4)P_2_. After the addition of E-IgG, the fluorescence around the engulfed E-IgG was monitored. In the control cells, PtdIns(3,4)P_2_ accumulated rapidly on the forming phagocytic cup. This accumulation peaked at 3 min and disappeared within 8 min after the start of phagocytosis (Fig 4A). In shInpp4a cells, PtdIns(3,4)P_2_ accumulated as rapidly as in the control cells, but its breakdown was markedly slowed (Fig 4A). The results suggest that Inpp4a hydrolyzes PtdIns(3,4)P_2_ on the phagosomal membrane. Similar results were obtained when EGFP-[PH(Akt)], which recognizes PtdIns(3,4)P_2_ and PtdIns(3,4,5)P_3_, was introduced to the cells (S2A Fig). We next monitored PtdIns(3)P, the product of Inpp4a, using EGFP-[3×FYVE(EEA1)] as a probe. As expected, the accumulation of PtdIns(3)P on the phagosome was decreased in the shInpp4a cells (Fig 4B). The impaired accumulation of phagosomal PtdIns(3)P was reproduced when the cells were challenged with zymosan particles (S2B Fig).

### Spatiotemporal localization of Inpp4a during cup formation

**Fig 5:**
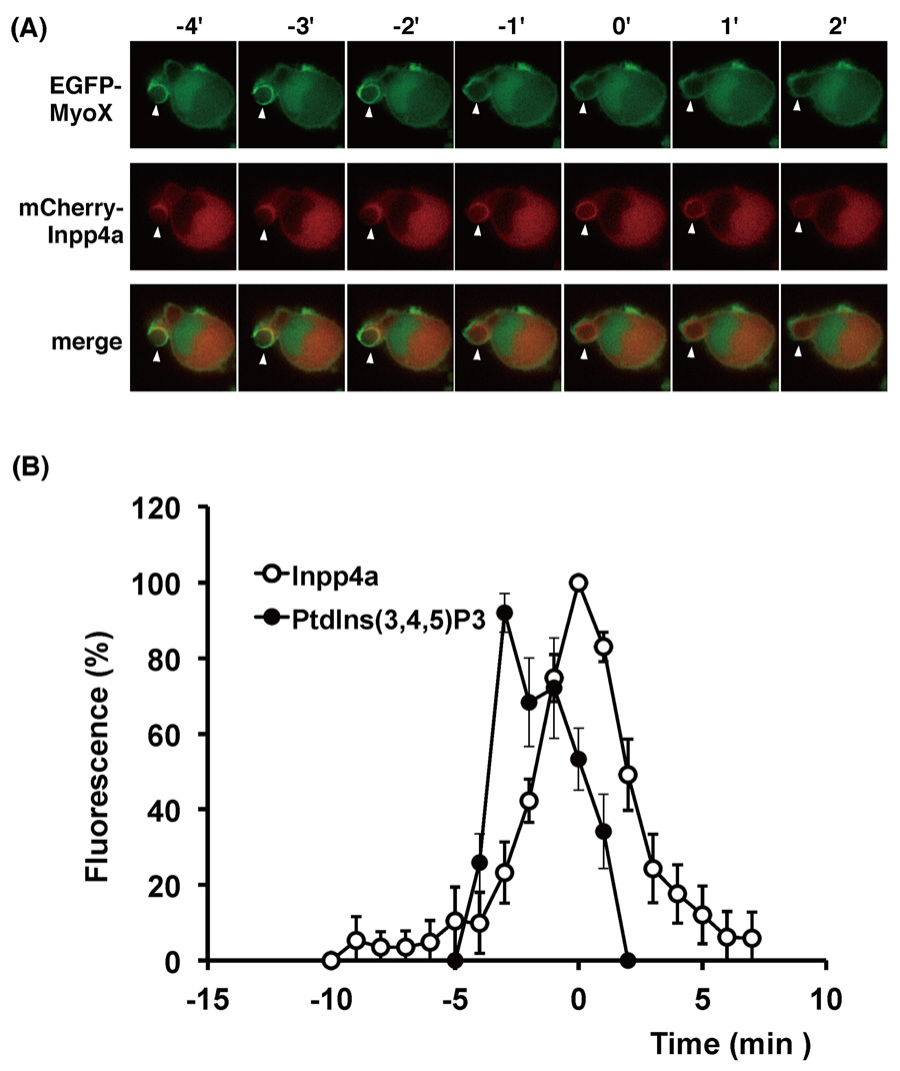
Timing of recruitment of Inpp4a and PtdIns(3,4,5)P_3_ during phagosome formation. (A, B) RAW264.7 cells were transfected with mCherry-Inpp4a along with EGFP-[PH1-PH2- PH1(MyoX)]. The cells were incubated with E-IgG at 37°C under a microscope. (B) The fluorescence intensity of each probe was quantified as described under "Materials and Methods" and shown as % of respective maximum values. The time course of the Inpp4a association is shown as a combined result from 12 different cells that were transfected with mCherry-Inpp4a (open circles), while that of PtdIns(3,4,5)P_3_ is calculated from 3 cells that were transfected with both mCherry-Inpp4a and EGFP-[PH1-PH2-PH1(MyoX)]. Time zero in the figures indicates the time when the mCherry-Inpp4a fluorescence around E-IgG peaked.

**Fig 6:**
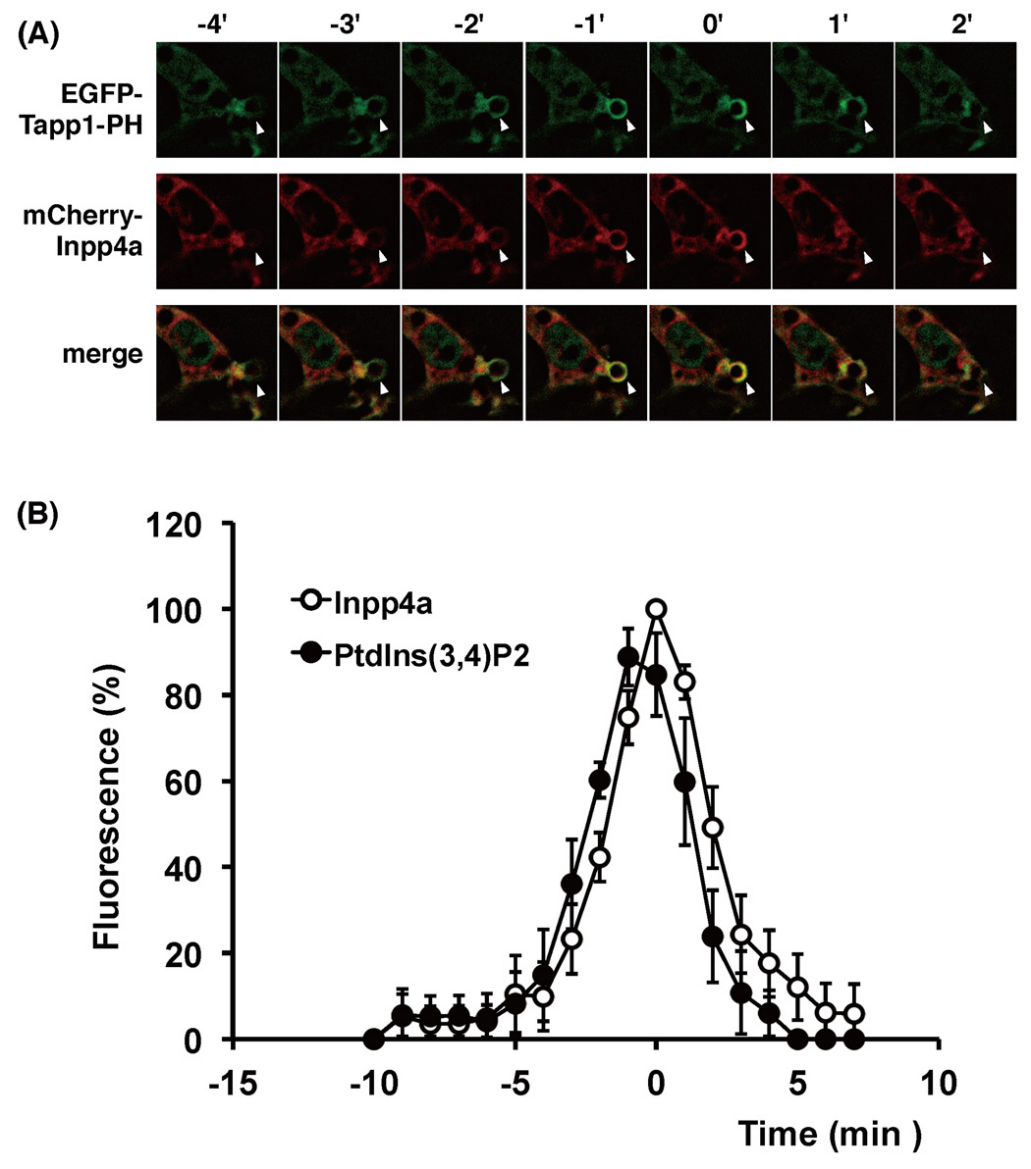
Timing of recruitment of Inpp4a and PtdIns(3,4)P_2_ during phagosome formation. (A, B) RAW264.7 cells were transfected with mCherry-Inpp4a along with EGFP-[2×PH (Tapp1)]. The cells were incubated with E-IgG at 37°C under a microscope. (B) The fluorescence intensity of each probe was quantified as described under "Materials and Methods" and shown as % of respective maximum values. The time course of Inpp4a was obtained as in Fig 5B, while that of PtdIns(3,4)P_2_ was calculated from 5 cells that were transfected with both mCherry-Inpp4a and EGFP-[2×PH (Tapp1)]. Time zero in the figures indicates the time when the mCherry-Inpp4a fluorescence around E-IgG peaked.

**Fig 7:**
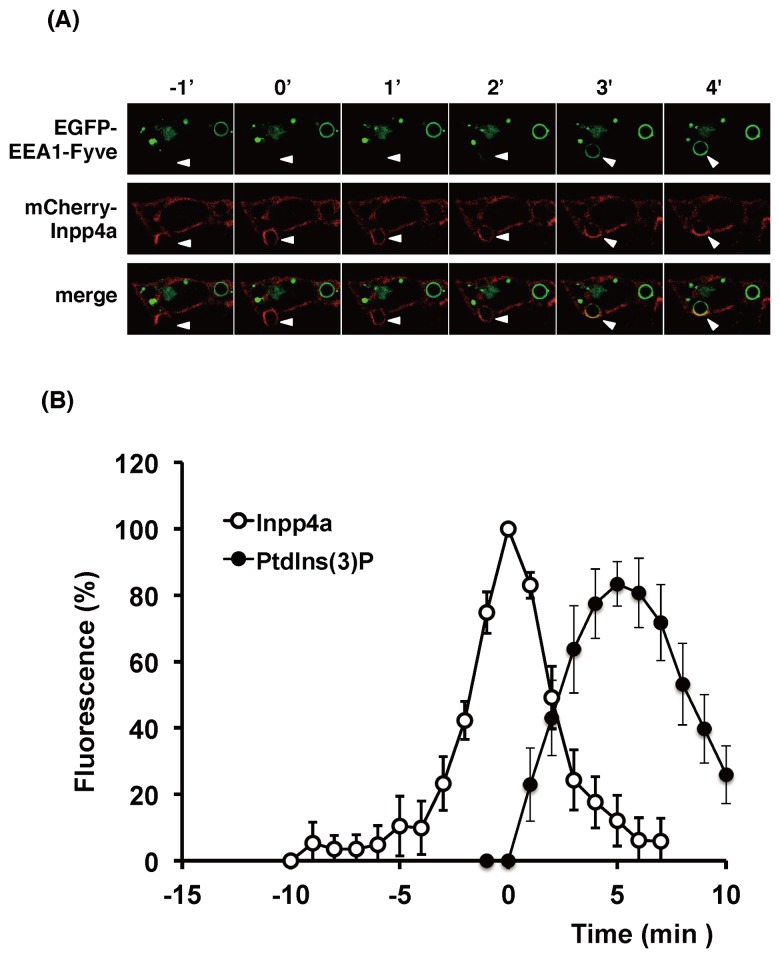
Timing of recruitment of Inpp4a and PtdIns(3)P during phagosome formation. (A, B) RAW264.7 cells were transfected with mCherry-Inpp4a along with EGFP-[3×FYVE (EEA1)]. The cells were incubated with E-IgG at 37°C under a microscope. (B) The fluorescence intensity of each probe was quantified as described under "Materials and Methods" and shown as % of respective maximum values. The time course of Inpp4a was obtained as in Fig 5B, while that of PtdIns(3)P was calculated from 9 cells that were transfected with both mCherry-Inpp4a and EGFP-[3×FYVE (EEA1)]. Time zero in the figures indicates the time when the mCherry-Inpp4a fluorescence around E-IgG peaked.

**Fig 8:**
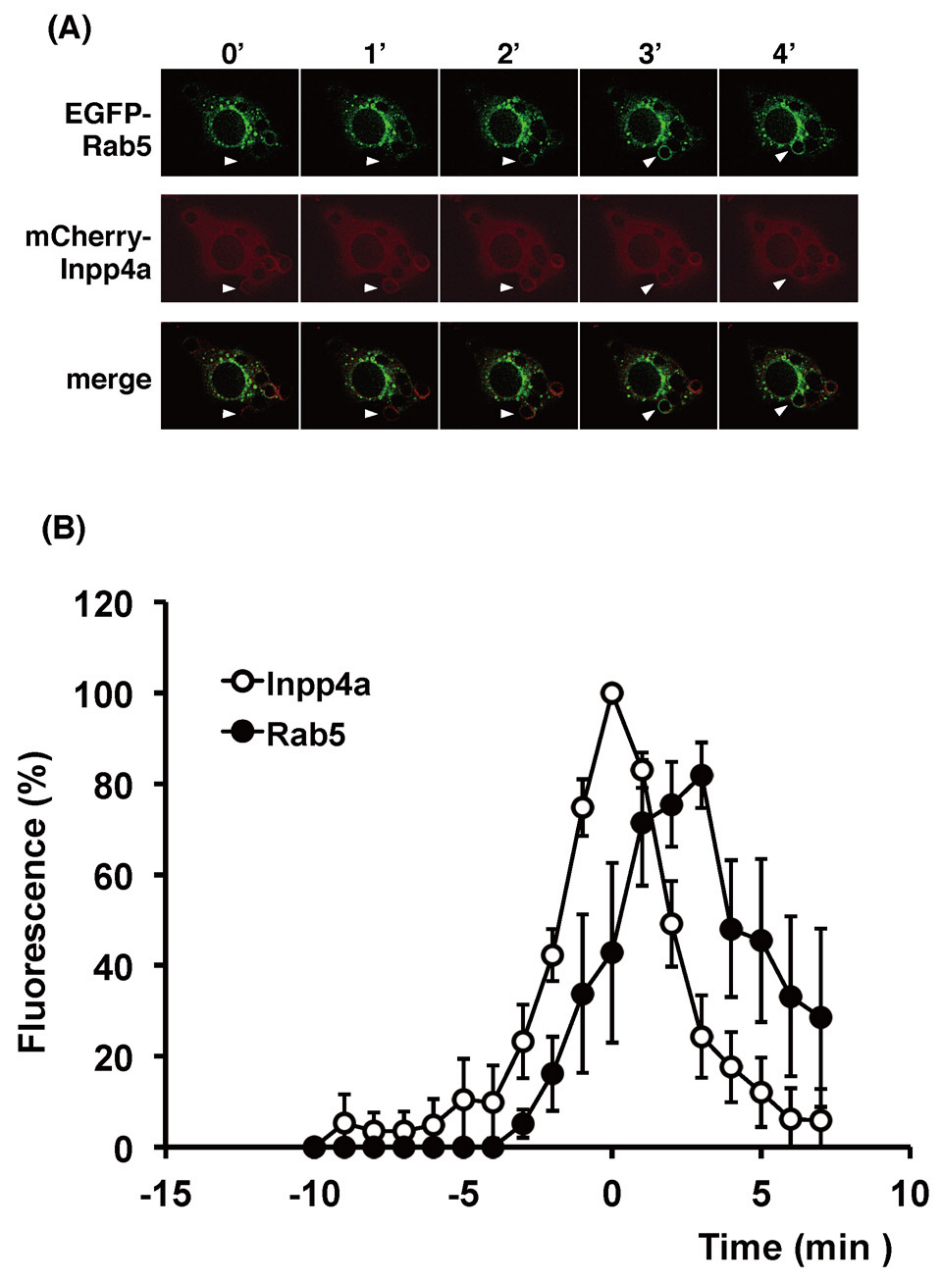
Timing of recruitment of Inpp4a and Rab5 during phagosome formation. (A, B) RAW264.7 cells were transfected with mCherry-Inpp4a along with EGFP-Rab5. The cells were incubated with E-IgG at 37°C under a microscope. (B) The fluorescence intensity of each probe was quantified as described under "Materials and Methods" and shown as % of respective maximum values. The time course of Inpp4a was obtained as in Fig 5B, while that of PtdIns(3)P was calculated from 5 cells that were transfected with both mCherry-Inpp4a and EGFP-[3×FYVE (EEA1)]. Time zero in the figures indicates the time when the mCherry-Inpp4a fluorescence around E-IgG peaked.

We next examined whether Inpp4a resides at the site of phagosome formation. Raw264.7 cells were transfected with mCherry-tagged Inpp4a and EGFP-[PH1-PH2-PH1(MyoX)], a specific probe for PtdIns(3,4,5)P_3_ [21], and then challenged with E-IgG (Fig 5A). As expected, the signals of PtdIns(3,4,5)P_3_ and Inpp4a were evident on the phagosome membrane. Interestingly, the full enclosure of the engulfed particle by PtdIns(3,4,5)P_3_ was attained earlier than that by mCherry-Inpp4a (at -2 and 0 min, respectively, in Fig 5A; time zero in Figs 5–8 indicates the time when the Inpp4a signal around E-IgG reached maximum). In Fig 5B, the time course of the Inpp4a association is shown (open circles) as a combined result from 12 different cells that were transfected with mCherry-Inpp4a. In the figure, the fluctuation of PtdIns(3,4,5)P_3_ is also shown (closed circles) as a combined result from 3 cells that were transfected with both mCherry-Inpp4a and EGFP-[PH1-PH2-PH1(MyoX)]. When the Inpp4a signal peaks (time zero), the intensity of PtdIns(3,4,5)P_3_ signal was significantly lower than its peak value (54±11%, n = 3, p<0.01). The result indicated that PtdIns(3,4,5)P_3_ peaks earlier than Inpp4a.

We then examined the spatiotemporal connection between Inpp4a and its substrate PtdIns(3,4)P_2_ on the phagosome (Fig 6). The accumulation of PtdIns(3,4)P_2_ was monitored by EGFP-[2×PH(TAPP1)]. There was no significant time-lag between the Inpp4a and PtdIns(3,4)P_2_ peaks (Fig 6B). It is intriguing to note, however, that the full enclosure of phagosome by PtdIns(3,4)P_2_ was attained a little earlier than that by mCherry-Inpp4a (at -3 and -2 min, respectively, in Fig 6A). This phenomenon was repeatedly observed, suggesting that Inpp4a recruitment follows the accumulation of PtdIns(3,4)P_2_. When Raw264.7 cells were transfected with mCherry-Inpp4a and EGFP-[3×FYVE(EEA1)], the full enclosure of the engulfed particle by Inpp4a was consistently observed earlier than that by its product PtdIns(3)P (at 1 and 4 min, respectively, in Fig 7A). The PtdIns(3)P peak was attained about 5 min later than the Inpp4a peak (Fig 7B).

### Irrelevance of Rab5 for the recruitment of Inpp4a

Rab5-GTP binds directly to and activates Inpp4a on the early endosome [11]. Thus, Rab5 is expected to mediate the recruitment of Inpp4a to the phagosome membrane. In the experiments shown in Fig 8, we examined the spatiotemporal relationship between Inpp4a and Rab5. Raw264.7 cells were transfected with mCherry-Inpp4a and EGFP-Rab5 and then challenged with E-IgG. Unexpectedly, mCherry-Inpp4a appeared around the engulfed particle far earlier than EGFP-Rab5 (at 0 and 3 min, respectively, in Fig 8A). When the Inpp4a signal peaks (time zero), the intensity of Rab5 signal was significantly lower than its peak value (40±17%, n = 5, p<0.01; Fig 8B). The result suggested that direct binding to Rab5 may not be the mechanism of Inpp4a recruitment to the phagosome membrane. Finally, we examined the possibility that Inpp4a on the phagosome membrane causes the Rab5 recruitment. To test the hypothesis, EGFP-Rab5 was transfected to shInpp4a cells, and the fluorescence was monitored. The result showed that Rab5 is normally recruited to phagosome even in the absence of Inpp4a (S3 Fig). Accordingly, Inpp4a and Rab5 may be independently recruited.

## Discussion

PI-metabolizing enzymes play inevitable roles in endocytic processes [5, 10]. In the present study, we observed that inositol polyphosphate-4-phosphatase type I (Inpp4a) is a negative regulator of phagocytosis. Raw264.7 cells that express shRNAs against Inpp4a (shInpp4a cells) showed a significant increase in phagocytic activity (Figs 2 and 3). This increase was completely abolished by the introduction of shRNA-resistant human Inpp4a (Fig 3C). Macrophages from Inpp4a knockout mice showed increased phagocytic activity (Fig 2B).

We have reported that the shRNA-based silencing of a class-IA PI3K, PI3Kα, markedly impairs the phagocytic activity of and phagosomal PtdIns(3,4,5)P_3_ accumulation in RAW264.7 cells [16]. In the present study, we showed that the PtdIns(3,4)P_2_ level increased while the PtdIns(3)P level decreased on the phagosome of shInpp4a cells (Fig 4, S2 Fig). Thus, the PtdIns(3,4,5)P_3_ that is formed on the phagocytic cup may be hydrolyzed to PtdIns(3,4)P_2_ by PI 5-phosphatases and then to PtdIns(3)P by Inpp4a. The role of Inpp4a in producing PtdIns(3)P has been described during the maturation of transferrin-containing vesicles in both HeLa and Cos7 cells [11, 14]. A direct interaction of Inpp4a with a small GTPase Rab5 has been proposed to play a key role in the PI turnover on clathrin-coated vesicles [11]. Similarly to this report, co-localization of Inpp4a and Rab5 can be observed on the phagosome of macrophages (Fig 8A). However, Inpp4a in the phagosomes may be recruited by a mechanism other than the direct interaction with Rab5 because Inpp4a appeared around the engulfed particle earlier than Rab5 (Fig 8). We observed that the time course of Inpp4a accumulation on phagosome is similar to that of PtdIns(3,4)P_2_ (Fig 6). Because the N-terminal C2 domain of Inpp4a is known to bind PtdIns(3,4)P_2_ [22], we speculate that this binding may be the basis of Inpp4a recruitment.

PI 5-phosphatases hydrolyzes PtdIns(3,4,5)P_3_ to produce PtdIns(3,4)P_2_, which in turn may recruit Inpp4a to phagosome. SH2-domain-containing 5-phosphatases, SHIP1 and SHIP2, are candidate enzymes that are responsible for this reaction, because these phosphatases directly bind to phagocytic receptors. They bind to ITIM domain of FcγRIIb and also to ITAM domain of FcγRI and DAP-12 [23, 24]. Inpp5b, OCRL and Inpp5e may also be involved in the reaction, because these enzymes are reportedly recruited to phagosome [25]. Inpp5b and OCRL bind to phagosome through Rab5 and APPL-1 [25]. Inpp5e binds to phagosome at the early stage of phagocytosis, and is proposed to induce the Rab5 recruitment [20].

Inpp4a is reportedly involved in the regulation of clathrin-mediated endocytosis [11, 14]. On the clathrin-coated vesicles of HeLa cells, a class-I_A_ PI3K, PI3Kβ, produces PtdIns(3,4,5)P_3_, which is metabolized to PtdIns(3,4)P_2_ by a 5-phosphatase Inpp5b and then to PtdIns(3)P by Inpp4a [11]. During the transferrin uptake by Cos7 cells, the phosphorylation of PtdIns(4)P by a class-II PI3K, PI3KC2α, produces PtdIns(3,4)P_2_, which is metabolized to PtdIns(3)P by Inpp4b [14]. On the phagosome of FcγRIIA-transfected fibroblasts, PtdIns(3)P is mostly produced by the phosphorylation of PtdIns. PtdIns 3-kinase (a sole member of class-III PI 3-kinase, Vps34) catalyzes this reaction [6]. In the present study, we observed that the PtdIns(3)P accumulation on the phagosome was significantly decreased in the shInpp4a cells (Fig 4B), indicating that Inpp4a produces a substantial amount of PtdIns(3)P in the phagosome of Raw264.7 cells. The recruitment of Inpp4a to the phagocytic cup began immediately after the appearance of PtdIns(3,4)P_2_ on the membrane (Fig 6). Interestingly, the Inpp4a level decreased before the accumulation of PtdIns(3)P peaked (Fig 7). The results suggested that the PtdIns(3)P production by the sequential dephosphorylation cascade may be operating mainly during the earlier stages of phagosome maturation before the PtdIns(3)P production by PtdIns 3-kinase is activated.

A rapid turnover of PtdIns(3,4)P_2_, the substrate of Inpp4a, is reported to control the maturation of clathrin-coated pits before fission, where the role of PtdIns(3,4)P_2_ has been considered to recruit sorting nexin 9 (SNX9) to the clathrin-coated vesicles [14]. In phagocytosis, the local fluctuation of PtdIns(4,5)P_2_ is known to control the phagosome formation via the effect on actin polymerization[26, 27]. Both the depletion and retention of PtdIns(4,5)P_2_ on the phagocytic cup are known to reduce phagocytosis [26, 28]. The acceleration of phagocytosis by class-I_A_ PI3K has been attributed to the production of PtdIns(3,4,5)P_3_ at the expense of PtdIns(4,5)P_2_. PtdIns(3,4,5)P_3_ is also proposed to promote phagosome closure via the recruitment of motor protein myosin X [18]. Thus, a disorder of PtdIns(4,5)P_2_ and PtdIns(3,4,5)P_3_ homeostasis due to the impaired sequential dephosphorylation system may explain the increased phagocytic activity of shInpp4a cells.

## Supporting Information

S1 Fig Augmentation of FcγR-mediated activation of Akt by depletion of Inpp4a. (A) shInpp4a cells (seq1) or (B) peritoneal macrophages from Inpp4a KO mice were stimulated with 30 μg/ml of aggregated IgG.
(TIFF)

S2 Fig Phagosomal levels of PtdIns(3,4)P_2_ (A) and PtdIns(3)P (B) in Inpp4a-deficient cells. (TIFF)

S3 Fig Normal recruitment of Rab5 to phagosome in Inpp4a-deficient cells. (TIFF)

S1 Table The primer pairs used to determine mRNA expression. (DOCX)
