# Supplementary figures and images for: Inositol Polyphosphate-4-Phosphatase Type I Negatively Regulates Phagocytosis via Dephosphorylation of Phagosomal PtdIns(3,4)P2
Source: PLoS One. 2015 Nov 4;10(11):e0142091. doi: 10.1371/journal.pone.0142091 (PMC4633150; doi:10.1371/journal.pone.0142091)

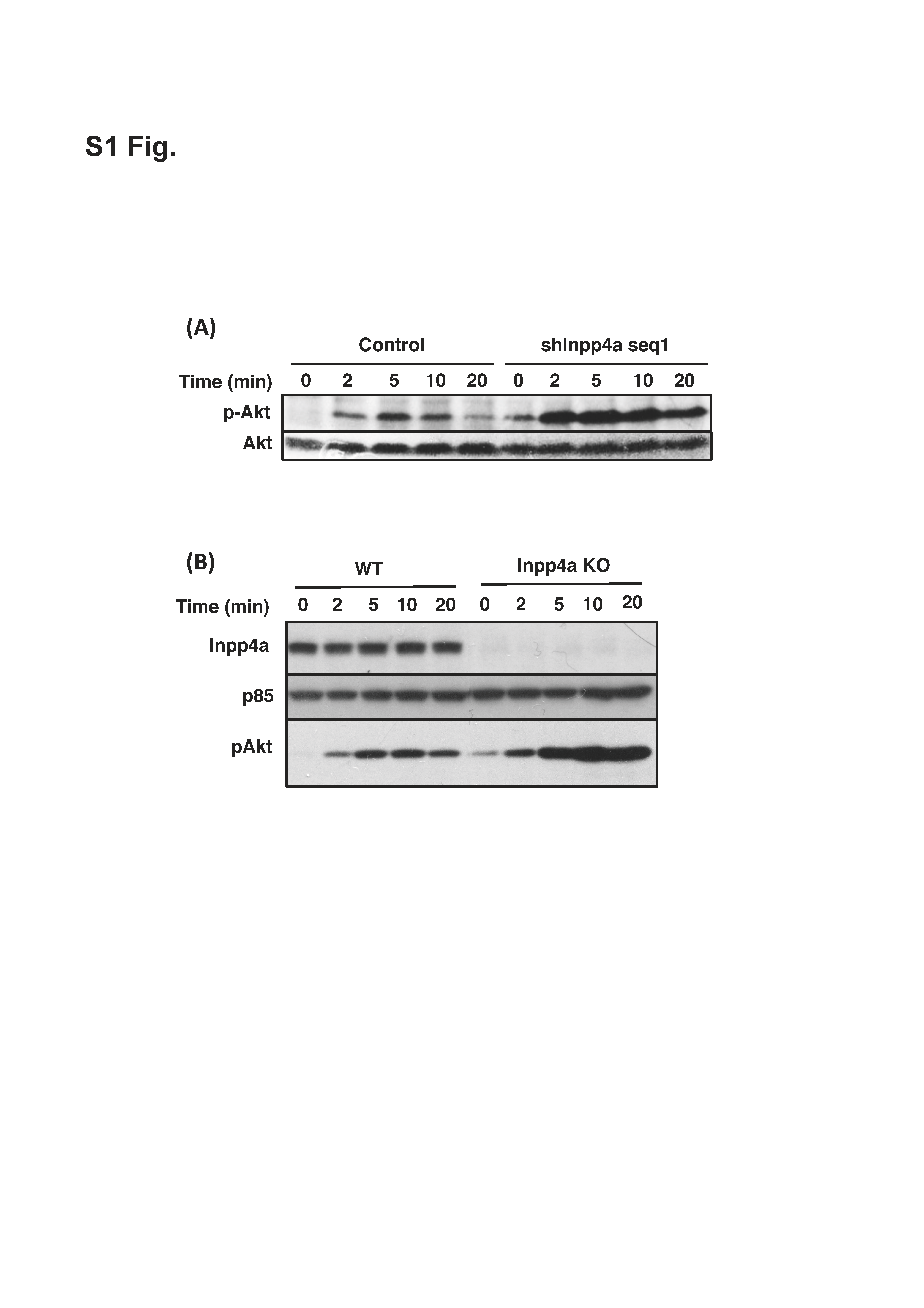

Supplement: S1 Fig — (A) shInpp4a cells (seq1) or (B) peritoneal macrophages from Inpp4a KO mice were stimulated with 30 μg/ml of aggregated IgG. (TIFF) [file pone.0142091.s001.tiff]

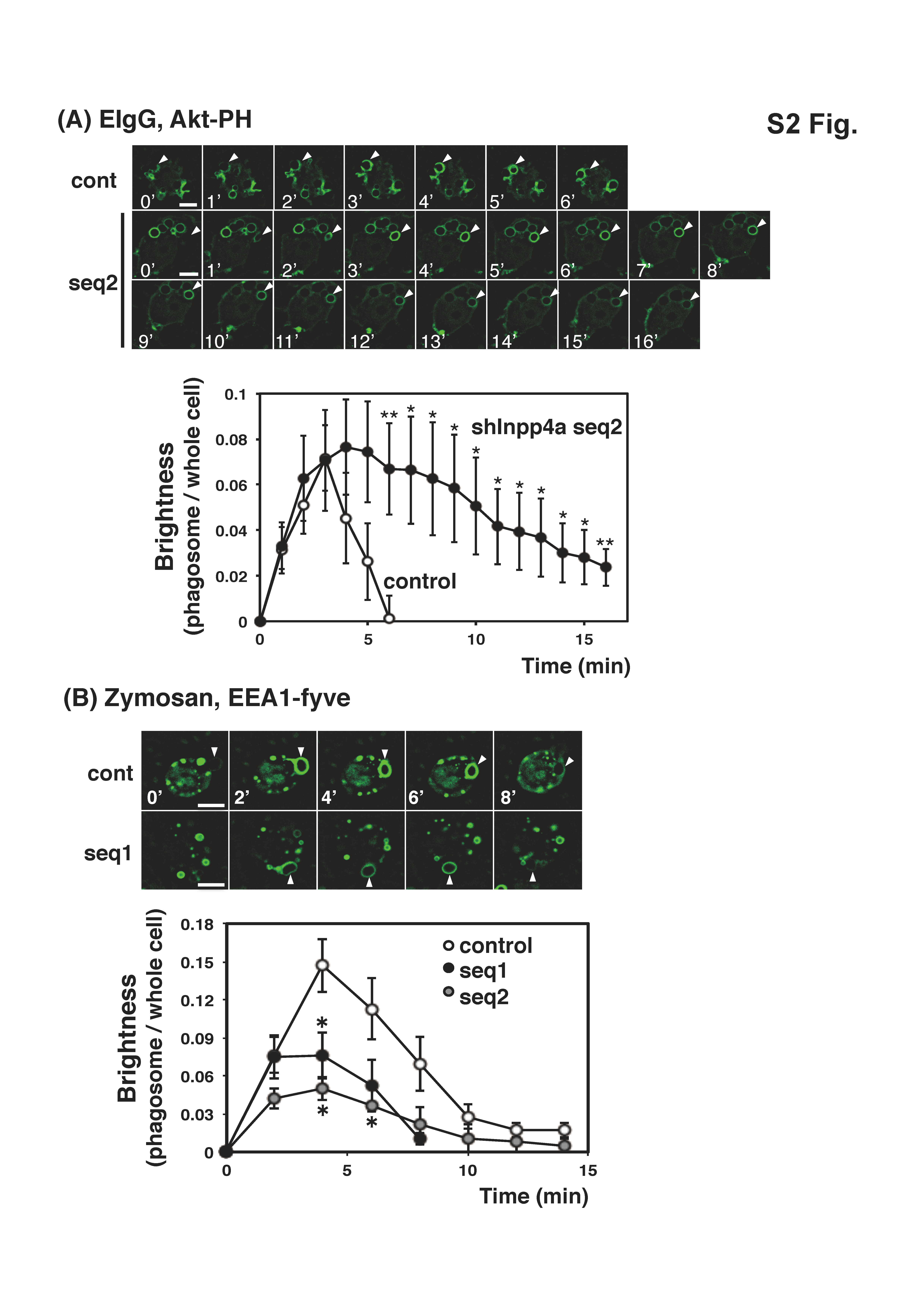

Supplement: S2 Fig — (TIFF) [file pone.0142091.s002.tiff]

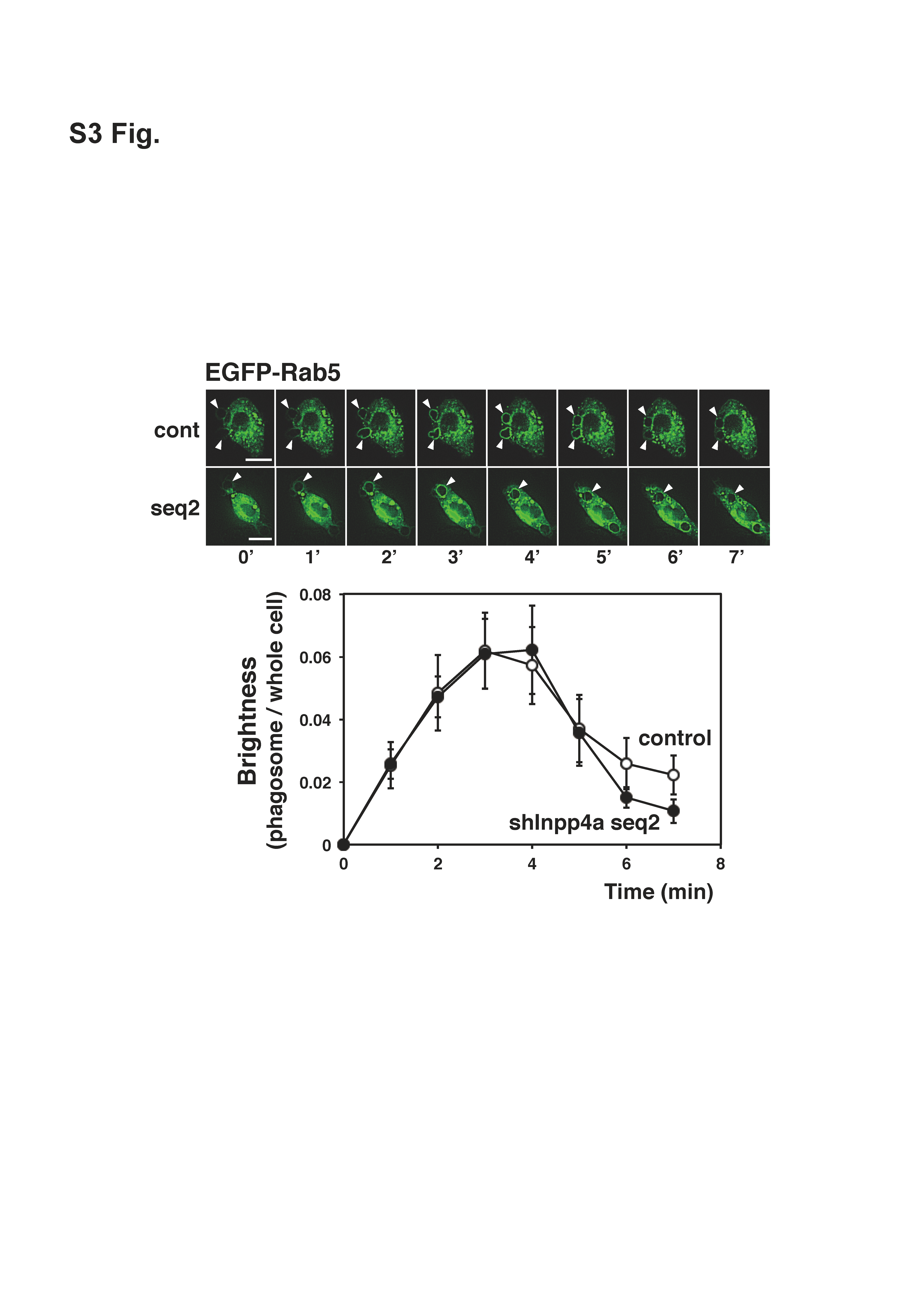

Supplement: S3 Fig — (TIFF) [file pone.0142091.s003.tiff]
