# Supplementary material for: Inositol Polyphosphate-4-Phosphatase Type I Negatively Regulates Phagocytosis via Dephosphorylation of Phagosomal PtdIns(3,4)P2
Source: PLoS One. 2015 Nov 4;10(11):e0142091. doi: 10.1371/journal.pone.0142091 (PMC4633150; doi:10.1371/journal.pone.0142091)
Supplement: S1 Table — (DOCX) [file pone.0142091.s004.docx]

**S1 Table**

**Primers for RT-PCR**

|  | Inpp4a | forward | 5’- GGTCTTCAGGACCGGACAGCAAC -3’ |  |
| --- | --- | --- | --- | --- |
|  |  | reverse | 5’- GACCACGTGGCATTGCGTCACA -3’ |  |
|  | Inpp4b | forward | 5’- GTGGATCATGGAAATCAAAGAGG -3’ |  |
|  |  | reverse | 5’- GTGGATCATGGAAATCAAAGAGG -3’ |  |
